# Effects and Mechanisms of Acupuncture Combined with Mesenchymal Stem Cell Transplantation on Neural Recovery after Spinal Cord Injury: Progress and Prospects

**DOI:** 10.1155/2020/8890655

**Published:** 2020-09-25

**Authors:** Huiling Tang, Yi Guo, Yadan Zhao, Songtao Wang, Jiaqi Wang, Wei Li, Siru Qin, Yinan Gong, Wen Fan, Zelin Chen, Yongming Guo, Zhifang Xu, Yuxin Fang

**Affiliations:** ^1^Research Center of Experimental Acupuncture Science, Tianjin University of Traditional Chinese Medicine, Tianjin 301617, China; ^2^Shanghai Minhang Traditional Chinese Medicine Hospital, Shanghai 201103, China; ^3^School of Traditional Chinese Medicine, Tianjin University of Traditional Chinese Medicine, Tianjin 301617, China; ^4^National Clinical Research Center for Chinese Medicine Acupuncture and Moxibustion, Tianjin 300381, China; ^5^Suzuka University of Medical Science, Suzuka 5100293, Japan; ^6^School of Acupuncture & Moxibustion and Tuina, Tianjin University of Traditional Chinese Medicine, Tianjin 301617, China

## Abstract

Spinal cord injury (SCI) is a structural event with devastating consequences worldwide. Due to the limited intrinsic regenerative capacity of the spinal cord in adults, the neural restoration after SCI is difficult. Acupuncture is effective for SCI-induced neurologic deficits, and the potential mechanisms responsible for its effects involve neural protection by the inhibition of inflammation, oxidation, and apoptosis. Moreover, acupuncture promotes neural regeneration and axon sprouting by activating multiple cellular signal transduction pathways, such as the Wnt, Notch, and Rho/Rho kinase (ROCK) pathways. Several studies have demonstrated that the efficacy of combining acupuncture with mesenchymal stem cells (MSCs) transplantation is superior to either procedure alone. The advantage of the combined treatment is dependent on the ability of acupuncture to enhance the survival of MSCs, promote their differentiation into neurons, and facilitate targeted migration of MSCs to the spinal cord. Additionally, the differentiation of MSCs into neurons overcomes the problem of the shortage of endogenous neural stem cells (NSCs) in the acupuncture-treated SCI patients. Therefore, the combination of acupuncture and MSCs transplantation could become a novel and effective strategy for the treatment of SCI. Such a possibility needs to be verified by basic and clinical research.

## 1. Introduction

Spinal cord injury (SCI) is a structural event with devastating consequences, such as permanent loss of motor, sensory, and autonomic functions and, in severe cases, paraplegia or quadriplegia below the level of injury [1]. The incidence of SCI tends to increase worldwide, with 17,000 new cases each year [2]. The pathophysiology of SCI can be categorized as primary injury and secondary injury. The secondary injury involves apoptosis and necrosis of damaged neurons, dislocation and demyelination of axons resulting from the loss of oligodendrocyte-derived myelin, local nerve inflammation caused by tissue edema or ischemia, and formation of a parenchymal cavity or glial scar in the spinal cord after hemorrhage [3, 4]. Currently, no effective treatment to reverse the trauma of SCI is available. This limitation is mostly due to the extremely limited capacity of the spinal cord to regenerate and enable the recovery of neurologic deficits [5]. The main strategies for the treatment of SCI include pharmacologic interventions, surgery, stem cell transplantation, behavioral therapy, physical stimulation, and supportive therapy. Among them, the current first-line treatment is the administration of high-dose corticosteroids, such as methylprednisolone sodium succinate, which can inhibit local inflammation and oxidative stress, protect the blood-spinal cord barrier, and prevent the death of neurons [6]. However, since multiple potential risks and equivocal clinical results have been reported, there is no consensus on the standardized application of corticosteroids in SCI treatment. Therefore, the development of safer and more effective therapies promoting neural restoration and functional recovery after SCI is of great clinical relevance.

Acupuncture is a procedure involving the insertion of a fine needle into the skin or deeper tissues at specific locations of the body (acupoints) to prevent and treat diseases [7]. Several lines of neuroanatomical and neurological evidence have demonstrated the abundant distribution of nerve endings in human meridians and acupoints, and the involvement of the nervous system is indispensable for the effects of acupuncture [8]. An increasing number of clinical studies have shown that acupuncture can effectively improve the functional recovery of neurons after various types of central nervous system injuries (CNSIs), including SCI [9]. The potential mechanisms mediating the effects of acupuncture include the prevention of inflammatory and oxidant stress, suppression of apoptosis, and stimulation of proliferation and differentiation of endogenous NSCs [10, 11]. However, there are still several obstacles to the application of acupuncture for the promotion of neural regeneration, such as an insufficient number of endogenous NSCs capable of differentiating into functional neurons. Thus, further research is needed to achieve progress in this field.

Mesenchymal stem cells (MSCs) are multipotential stem cells derived from the mesoderm. They are capable of self-renewal and multilineage differentiation and maintain these biological characteristics after large-scale expansion *in vitro*. MSCs have been regarded as pluripotent “seed cells” with two main therapeutic effects. One effect is the migration of cells to the damaged tissue and differentiation into tissue-specific cell types, thus restoring the function of target tissues and organs. The other effect is the inhibition of local inflammation, apoptosis, and fibrosis; promotion of angiogenesis; and stimulation of regeneration and differentiation of resident tissue progenitor cells by secreting soluble growth and trophic factors [12, 13]. Moreover, MSCs have several advantages, such as the ability to differentiate into multiple lineages, low immunogenicity, abundant sources, simplicity of preparation, and low tumorigenicity [14]. Many clinical and basic studies have documented that while MSC transplantation is effective for SCI, it is associated with certain problems, such as unpredictable cell viability, low efficiency of differentiation into corresponding tissue cells, and insufficient ability to migrate to target organs [15–17]. In recent years, studies in China and abroad have demonstrated that the combination of acupuncture and MSC transplantation provides a greater benefit in SCI patients than either procedure alone. Therefore, the present analysis addresses the clinical efficacy and potential mechanisms of acupuncture and acupuncture combined with MSC transplantation in the treatment of SCI, utilizing the data generated during the past 20 years. The objective of this work was to critically evaluate the underlying evidence and provide novel insights for the clinical application of acupuncture in SCI therapy.

## 2. The Effect of Acupuncture on Neural Restoration in Spinal Cord Injury and Its Mechanism

### 2.1. Clinical Efficacy of Acupuncture in Neurological Rehabilitation of SCI Patients

Several randomized controlled trials have demonstrated that different acupuncture methods can improve the sensory and motor function of SCI patients (Table 1). Pooled analyses in a meta-analysis showed that acupuncture had a beneficial effect on neurological recovery (relative risk: 1.28, 95% confidence interval (CI): 1.12-1.50), motor function (weighted mean difference: 6.86, 95% CI: 0.41-13.31), and functional recovery (standardized mean difference: 0.88, 95% CI: 0.56-1.21). Moreover, acupuncture improved the activity of daily living (ADL) in SCI patients, particularly if applied at the back of the Governor Vessel (GV) and bladder channel acupoints [9, 18]. Wong et al. [19] performed an RCT evaluating the efficacy of acupuncture in 100 patients with SCI and demonstrated that acupuncture implemented early in acute SCI increased Functional Independence Measure scores. Wang et al. [20] conducted a prospective RCT with 48 SCI patients to compare the efficacy of paraplegia-triple-needling method (GV and the Back-shu) and the conventional acupuncture at GB30 (*Huantiao*), ST36 (*Zusanli*), GB39 (*Xuanzhong*), and SP6 (*Sanyinjiao*). The results indicated that both therapies improved the ADL score and the comprehensive function in patients with traumatic SCI of the thoracic and lumbar vertebrae. The paraplegia-triple-needling combined with the rehabilitation training provided a better long-term improvement [20]. Also, it has been reported that acupuncture can effectively ameliorate various complications of SCI, such as pain, neurogenic bladder, pressure sores, spasm, and osteoporosis [2, 21–23]. However, the above-indicated meta-analysis identified several limitations of the performed studies, such as the lack of high-quality multicenter large-size trials, the lack of uniform acupuncture methods, the bias of clinical trials, and the incidence of adverse events caused by acupuncture [18, 24]. Therefore, the standardization of acupuncture procedures may facilitate the evaluation of their efficacy and clinical outcomes.

### 2.2. Neuroprotective and Neurogenerative Mechanisms of Acupuncture in SCI

The pathological processes after SCI can be divided into three stages: acute, subacute, and chronic. The first stage includes a local inflammatory response, which mainly involves infiltration of immune cells such as macrophages, T lymphocytes, neutrophils, and microglia, and the release of proinflammatory cytokines, such as tumor necrosis factor-*α* (TNF-*α*), interleukin-1*β* (IL-1*β*), and interleukin-6 (IL-6). At the mitochondrial level, insufficient reduction of oxygen and nitrogen molecules generates high levels of reactive oxygen species (ROS) and reactive nitrogen species (RNS), respectively. ROS and RNS trigger neuronal DNA damage and oxidative stress-induced cell death [25]. Additionally, the activation of astrocytes leads to the deposition of a high amount of the extracellular matrix, inhibiting cell migration and axon growth and repair, and forms a large cystic cavity in the injured region. Together, these mechanisms contribute to the progressive damage of the primary injured tissue, producing a “secondary injury”. The secondary injury is followed by the subacute phase, which lasts for approximately 1 year after the initial event. During the subacute phase, various factors lead to a further expansion of the injured area and the development of the chronic stage [26]. In addition, these complex pathological changes engender several complications, such as respiratory and cardiac dysfunction, abnormal temperature control, hypo- and hypertension, neurogenic bladder, and sexual dysfunction [22].

In the past 20 years, the mechanism of the action of acupuncture on SCI has been extensively studied using standardized acupuncture methods, such as electroacupuncture (EA) (Table 2). The most frequently used acupoints include the GV and bladder channel acupoints, such as Ex-B05 (*Jiaji* acupoints), GV14 (*Dazhui*), GV4 (*Mingmen*), and a few other meridian acupoints, such as ST36. When acupuncture is applied to the back acupoints, the needle is directed mostly toward the dura mater, indicating that EA may act directly on the meningeal branches of the spinal cord at the corresponding nerve segments, including the spinal dura mater, vertebra, dura mater, and ligaments [27]. The neural plasticity defines the ability of the nervous system to repair itself, structurally and functionally. Acupuncture provides a kind of physical peripheral stimulation and central sensory feedback to promote functional recovery, which could be essential for the formation of new synapses after SCI [27]. The potential mechanisms by which acupuncture modulates the neural plasticity and promotes neural restoration and functional recovery are summarized below.

#### 2.2.1. Neuroprotective Effect of Acupuncture

SCI causes the loss of a large number of neurons, oligodendrocytes, astrocytes, and microglia, leading to various functional disorders. Therefore, a timely and effective prevention of nerve cell death is critical for the treatment of SCI [28]. It has been demonstrated that acupuncture provides neuroprotection by inhibiting oxidative stress and inflammatory response after SCI. Juarez Becerril et al. [29] reported that EA stimulation of GV4 reduced the level of ROS by 15%, decreased the extent of spinal cord tissue damage by 25%, and improved the motor function of hindlimbs in paralyzed rats by 18.1%. Jiang et al. [30] demonstrated that EA of GV26 (*Shuigou*) and GV16 (*Fengfu*) reduced the synthesis and release of proinflammatory factors such as TNF-*α*, IL-1, and IL-6 in the damaged area of acute SCI in rats. Moreover, EA at GV6 (*Jizhong*) and GV9 (*Zhiyang*) not only reduced the population of M1 macrophages and the expression of their marker CD86 and associated cytokines TNF-*α*, IL-1*β*, and IL-6 but also increased the proportion of M2 macrophages and upregulated the expression of their marker CD206 and released cytokine IL-10, indicating that EA could promote macrophage polarization from proinflammatory M1 phenotypes to anti-inflammatory M2 phenotypes. Moreover, M2 macrophage polarization induced the synthesis and secretion of neurotrophic factor-3 (NT-3) that has a neuroprotective activity [31]. Choi et al. [32] showed that EA at GV26 and GB34 (*Yanglingquan*) in rats with acute SCI inhibited the apoptosis and demyelination of spinal cord neurons. The mechanism of this effect involves the suppression of inflammation induced by the activation of microglia through the downregulation of p38 mitogen-activated protein kinase (MAPK) phosphorylation. Apoptosis signaling involves endogenous pathways mediated by mitochondria and exogenous pathways mediated by death receptors. The endogenous pathway is activated by the change in mitochondrial membrane permeability, the release of proapoptotic molecules such as cytochrome c into the cytoplasm, and the activation of caspase-9 cascade. Conversely, the exogenous pathway is initiated by the stimulation of caspase-8 after the apoptotic signal activates death receptors FAS, TRAIL-Rs, and TNF receptor 1 and the related death domain [33–35]. These two pathways eventually converge at caspase-3, which executes apoptosis by cleaving the cytoskeleton and activating DNA-degrading enzymes. Du et al. [36] documented that the penetrating acupuncture at BL54 (*Zhibian*) and ST28 (*Shuidao*) inhibited exogenous death receptor-mediated apoptosis of neurons in acute SCI and downregulated the local expression of FAS and caspase-3. Shi et al. [37] showed that the elongated needle therapy at BL54 and ST28 promoted the recovery from SCI. This beneficial effect was associated with the suppression of inflammation via the phosphoinositide 3-kinase/Akt (PI3K/Akt) and MAPK/extracellular signal-regulated kinase (ERK) signaling pathways, which resulted in the downregulation of the Bax protein, upregulation of Bcl-2, and inhibition of the mitochondria-mediated apoptosis. Therefore, acupuncture may promote the survival of neurons after SCI by blocking both the endogenous and exogenous apoptosis pathways, facilitating the activation of SCI repair and functional recovery [38].

#### 2.2.2. Acupuncture Modulates Neural Plasticity and Promotes Neural Regeneration

A growing body of evidence indicates the crucial role of intracellular signaling cascades, such as the Wnt, Notch, and ROCK pathways, in neural plasticity and regeneration after SCI. Thus, the development of therapeutic agents targeting these pathways is expected to contribute to the treatment of SCI.

The Wnt signaling pathway plays an important role in the proliferation, differentiation, and axon orientation of NSCs. Wnt-1, the key element in the Wnt pathway, and the critical transcription factor *β*-catenin are highly expressed in the early stage of SCI, which is consistent with the reactive proliferation of endogenous NSCs of the spinal cord [39, 40]. Xu et al. [40] demonstrated that a fire needle at the Ex-B05 points promoted lower limb locomotor function in SCI rats. Moreover, they documented that the potential mechanism underlying the effect of acupuncture involves the stimulation of proliferation and differentiation of NSCs into neurons by the activation of the Wnt/*β*-catenin pathway (Wnt-3a, GSK3, *β*-catenin, and ngn1) and inhibition of the overexpression of MAPK-ERK kinase/extracellular signal-regulated protein kinases 1 and 2 (ERK1/2) and cyclin D1. Wang et al. [39] found that the expression of Wnt-1, Wnt-3a, and *β*-catenin in the injured area was increased at 1, 7, and 14 days after SCI, while the expression of Wnt-1, Wnt-3a, and *β*-catenin was increased by EA at GV14 and GV4. These results suggest that GV EA may promote the regeneration of neurons by activating the Wnt/*β*-catenin signaling pathway. The above studies only mentioned that acupuncture improves locomotor function as well as regulates these pathway proteins, where changes expressed by NSCs need to be clarified, and additional supporting data generated by the loss-of-function methodology are needed to reach a definitive conclusion.

Notch signaling is a classical pathway controlling the proliferation and differentiation of endogenous NSCs. There are four types of Notch receptors, named Notch1 through Notch4; their ligands are members of the Delta/Serrate/Lag2 protein family, such as Delta. The activation of Notch receptors induces the transcription and expression of downstream repressor genes, such as Hes 1 and Hes 5, which regulate cell proliferation and differentiation [41]. After SCI, the Notch signaling is activated, stimulating endogenous NSC proliferation and differentiation predominantly into astrocytes, hindering SCI repair. Geng et al. [42] documented that EA at GV14 and GV4 promoted the proliferation of endogenous NSCs in the spinal cord and inhibited the local expression of Notch1, Notch3, and Notch4, preventing endogenous NSCs from differentiating into astrocytes. It has also been shown that EA at GV and the bladder channel in SCI rats inhibited Notch signaling and increased the number of BrdU/neuron-glia antigen 2 (NG2) double-positive cells around SCI. Additionally, this procedure promoted the proliferation of endogenous NSCs and the differentiation of oligodendrocytes in the injured spinal cord [43].

Rho/ROCK signaling is mainly responsible for regulating cytoskeleton organization, cell growth, cell migration, proliferation, and development [44]. The RhoA/ROCK pathway mediates the effects of myelin-associated axon growth inhibitors (Nogo), myelin-associated glycoprotein, oligodendrocyte-myelin glycoprotein, and repulsive guidance molecule. Blocking RhoA/ROCK signaling reverses the inhibitory effects of these molecules on axon outgrowth and promotes axonal sprouting and functional recovery in CNSI models [45]. Wu et al. [46] demonstrated that EA treatment at GV14, GV4, SP6, GB30, ST36, and BL60 (*Kunlun*) for 7 days improved tissue repair and neurological functional recovery, reduced neuronal apoptosis, and suppressed the expression of RhoA and Nogo-A at the SCI lesion. It has also been shown that EA downregulated the expression of RhoA, ROCK II, myosin light chain, Nogo-A, NgR, and LINGO-1 in the anterior horn of the spinal cord, resulting in an improvement of the motor function of the hindlimb in SCI rats [47–49]. These data suggest that acupuncture can improve SCI neural restoration by enhancing the Rho/ROCK signaling. However, the specific mechanism underlying the effects of acupuncture on axon growth and regeneration mediated by the Rho/ROCK signaling has not been fully elucidated. Whether the regulation of local inflammation and cell migration by acupuncture involves this signaling pathway remains to be determined.

Endogenous neurotrophic factors (NTFs), such as the nerve growth factor (NGF) [50], brain-derived neurotrophic factor (BDNF), and NT-3 [51, 52], act by binding to their receptors, respectively, TrkA, TrkB, and TrkC [53]. These factors are essential to promote axon sprouting and neuronal regeneration in the injured site. NGF/tropomyosin receptor kinase A (TrkA) signaling can prevent apoptosis by the activation of the PI3K/Akt pathway. Acupuncture has been shown to increase the expression of the BDNF receptor kinase B (TrkB) [54] by activating tropomyosin through the PI3K/Akt and ERK1/2 signaling. These processes lead to the phosphorylation and activation of the cyclic AMP (cAMP) response element-binding transcription factor, which upregulates the transcription of the BDNF gene [54]. In addition to affecting NTFs and their receptors, acupuncture can modulate neural plasticity by inhibiting the expression of the epidermal growth factor receptor [55] and glial fibrillary acidic protein (GFAP) [56] in the spinal cord, thus promoting axon regeneration and preventing the formation of the glial scar.

In summary, the current researches on the mechanism of acupuncture in SCI are focused mostly on the level of single molecules and/or signaling pathways. However, a wide range of interactive communication exists between different signaling pathways, and acupuncture may regulate a complex network of multiple signaling molecules and pathways. This notion is consistent with the holistic regulation characteristics of acupuncture, involving multiple targets, links, approaches, and levels. How acupuncture affects this complex network requires further investigation. Moreover, due to the small number of endogenous NSCs and the unfavorable microenvironment of the injured region of SCI, the proliferation and differentiation of endogenous NSCs may not be sufficient to replace the damaged central nervous system. Thus, it is crucial to identify treatments that could be combined with acupuncture to achieve a better promotion of the restoration of neurons after SCI.

## 3. The Effects of Acupuncture Combined with Mesenchymal Stem Cells on SCI and Their Mechanism

### 3.1. Effects of Mesenchymal Stem Cells on Neural Restoration after SCI and Their Mechanisms

MSCs are important members of the stem cell family; they are derived from the early developmental mesoderm and belong to pluripotent stem cells [61]. Given their strong proliferation ability and multilineage differentiation potential, MSCs can be induced to generate neurons and glial cells [15, 62]. Clinical studies have confirmed that MSC transplantation is effective in the treatment of post-SCI dysfunction [14]. In the first longitudinal study of the effect of MSCs on the outcomes in SCI patients, autologous MSCs were isolated from each patient's bone marrow, amplified, and implanted by intramedullary or intradural injection [63]. Within 6 months after implantation, motor function was significantly improved in 7 of 10 patients. After 3 years of follow-up, motor function continued to improve, and no other complications or signs of tumor formation were present [64]. Similarly, a recent clinical trial showed that in 10 of 14 SCI patients, the treatment with MSCs ameliorated the sensory impairment, as documented by the improvement in the American Spinal Injury Association (ASIA) motor and sensory scores [65]. Recently, clinical trials of MSC transplantation for the treatment of acute and subacute SCI patients have been systematically reviewed, and the conclusion was reached that this therapy can safely and effectively improve SCI-related symptoms such as dyskinesia [66, 67]. A retrospective study of acute SCI showed that 19 (70%) of the completed (*n* = 18) and ongoing (*n* = 9) clinical trials were focused on the intrathecal injection of MSCs for the treatment of SCI [66]. However, the exploration of other transplantation methods was also underway and will provide a clinical basis for the optimal route of MSC transplantation for the treatment of SCI. Moreover, it is generally considered that to improve the survival rate of MSCs, the best time window for transplantation is within 1-2 weeks after the injury [68]. Also, implantation of MSCs results in a short-term improvement of autonomic nerve function and relieves from sweat gland secretion disorder and orthostatic hypotension, i.e., goals that could not be achieved by the traditional treatment.

The mechanisms underlying the effects of MSC transplantation in the treatment of SCI include the activation of multiple paracrine or autocrine NGFs, neuron regeneration, nerve loop reconstruction, integration of transplanted cells and host cells, and prevention or reduction of glial scar formation at the site of injury [66]. After migrating to the lesion, implanted MSCs can differentiate into functional neurons, which can form synapses with host neurons [68]. They also can improve axonal regeneration, inhibit demyelination while promoting myelin regeneration [16, 69–72], and reconstruct functional neural networks [15]. It has been proposed that the therapeutic action of implanted MSC in SCI is based on the secretion of a variety of factors, such as NGF, NT-3, and BDNF [73, 74]. Furthermore, paracrine immunomodulatory mediators secreted by MSCs can reduce harmful inflammation by inhibiting the differentiation of macrophages and microglia into neurotoxic, proinflammatory M1 subsets and promoting the generation of immunomodulatory M2 subsets which contribute to axonal growth and myelin regeneration [75]. The paracrine factors also help to promote the differentiation of MSCs, creating an environment facilitating the survival of transplanted MSCs, axonal regeneration, and integration of implanted cells with host cells.

In summary, MSC transplantation appears to represent an effective treatment for SCI patients, but large-scale phase III clinical trials are needed. The mechanism of the beneficial effects of MSCs involves neuroprotection, immune regulation, neuron regeneration, and the restoration of nerve conduction. Together, these processes contribute to structural repair and functional recovery of the injured spinal cord. However, some studies have shown that MSCs located in the lesion cannot differentiate into neurons due to an unfavorable microenvironment in the injured spinal cord and their low survival rate. Therefore, an improvement in the survival and directional differentiation of MSCs is essential to achieve progress in clinical applications of these cells in SCI treatment.

### 3.2. Effects and Mechanisms of the Combined Acupuncture/MSC Therapy for SCI

In recent years, extensive research has been performed, and some progress has been achieved, on the efficacy and mechanisms of acupuncture combined with MSC transplantation in the treatment of acute CNSIs such as SCI, traumatic brain injury, stroke, and cerebral palsy [76]. The combination of acupuncture and MSC transplantation resulted in an improvement of the SCI comprehensive functional score and the BBB motor score. Importantly, the curative effect of the combination therapy was better than that of either acupuncture or MSC implantation alone (Table 3).

The mechanism responsible for the effect of the combined therapy appears to depend on the promotion of the survival and differentiation of MSCs. Ding et al. [77] documented that 10 weeks of combination therapy increased the formation of descending corticospinal tract projections into the lesion and showed improved Basso-Beattie-Bresnahan (BBB) scores and enhanced motor-evoked potentials in rats with spinal cord transection. Sun et al. [78] have shown that the combination therapy increased the expression of neuron- and glial-specific markers (neuron-specific enolase (NSE) and GFAP, respectively) more than MSC transplantation alone, suggesting that acupuncture promotes the differentiation of MSCs into neurons and glial cells. The structural and functional recovery after the combination treatment may also be due to the downregulation of expression of GFAP and chondroitin sulfate proteoglycans (CSPGs), which could prevent axonal degeneration and improve axonal regeneration.

The neurotrophic factor NT-3 has an important function in the development, differentiation, and survival of neurons and in signal transduction. NT-3 also induces the growth of axons from the intact corticospinal tract across the midline to the innervated side [79, 80]. Liu et al. [81] documented that the combination treatment increased the number of surviving MSCs, an effect that may be related to the acupuncture-induced increase in the cAMP level in the SCI area. cAMP, in turn, can increase the expression of endogenous NT-3, promoting the differentiation of MSCs into neuron-like cells and oligodendrocytes; these cells replace the injured tissue and fill the cystic area [82–84]. Ding et al. [85] grafted TrkC (NT-3 receptor)-modified MSCs (TrkC-MSCs) into the demyelinated spinal cord and applied EA. In this experiment, EA increased NT-3 expression, promoting the differentiation of TrkC-MSCs into oligodendrocyte-like cells, remyelination, and functional improvement of the demyelinated spinal cord. Additional effects of acupuncture involve the inhibition of GFAP secretion, promotion of the synthesis of laminin, and regeneration of calcitonin gene-related peptide-positive and serotonin-positive nerve fibers and corticospinal tract nerve fibers. Also, acupuncture reduces the size of the nerve cavity to prevent further expansion of the nerve scar and creates a favorable microenvironment for nerve fiber regeneration and penetration into the injured area. Ultimately, these effects can lead to an improvement in motor function. Moreover, acupuncture can enhance the migration of MSCs by increasing the phosphorylation of Akt and ERK. Finally, unpublished data obtained in our laboratory showed that the expression of chemokines (such as CXCL1) and their receptors (such as CXCR2) in target organs increased significantly after acupuncture. We have raised the possibility that the chemotactic effect of acupuncture may enhance the homing ability of MSCs, which is critical for the targeted migration of these cells.

In summary, we advance a hypothesis that the biological mechanism underlying the beneficial impact of acupuncture combined with MSCs transplantation involves the improvement in the local microenvironment at the site of injury through the neuroprotective and immunomodulatory effects of acupuncture. The combination therapy can improve the survival rate and direct the differentiation of MSCs, promoting the differentiation of exogenous MSCs into oligodendrocyte-like or neuron-like cells. Secondly, the combined treatment promotes targeted migration of MSCs to the spinal cord. Thirdly, transplanted MSCs can release a large amount of neurotrophic and immunomodulatory factors that, through paracrine mechanisms, can enhance acupuncture neuroprotection, nutrition, and axonal budding, counteracting problems such as the small number of host endogenous NSCs and the limited ability of acupuncture to promote their differentiation.

## 4. Conclusion

**Figure 1:**
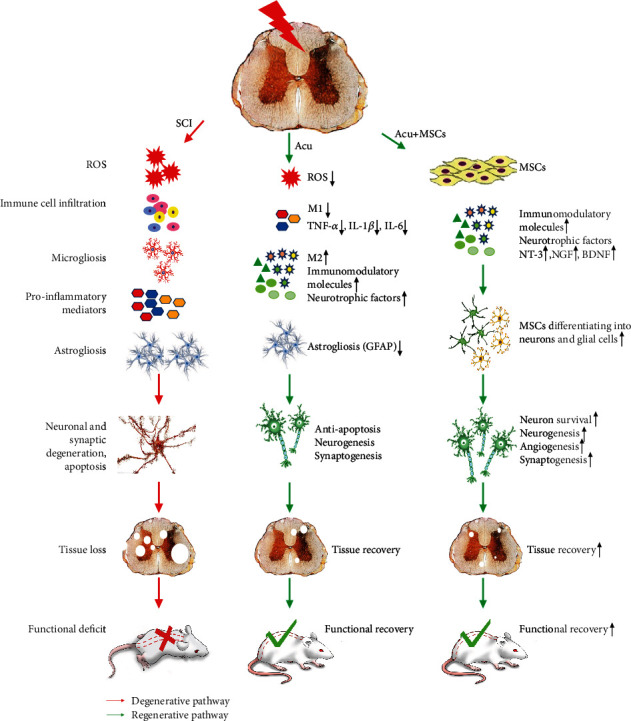
Effects of the combination of acupuncture and MSC transplantation on neural recovery after spinal cord injury (SCI) and the underlying mechanisms. ROS: reactive oxygen species; TNF-*α*: tumor necrosis factor-*α*; IL-1*β*: interleukin-1*β*; IL-6: interleukin-6; MSCs: mesenchymal stem cells; GFAP: glial fibrillary acidic protein; NT-3: neurotrophic factor-3; NGF: nerve growth factor; BDNF: brain-derived neurotrophic factor.

In conclusion, the recovery of patients after SCI is difficult due to the complex pathological sequelae of the injury and limited regenerative capacity of neurons (Figure 1). Acupuncture is effective for SCI-induced neurologic deficits. The potential mechanisms of acupuncture actions involve the protection of neurons against inflammation, oxidation, and apoptosis and the improvement of the local microenvironment. Additionally, acupuncture can promote neural regeneration and axon sprouting via multiple cellular signal transduction pathways, such as ROCK, Wnt, and Notch. Although MSC transplantation alleviates neural deficits and related complications, low survival and differentiation rates of MSCs limit the effects of their use in SCI. Several studies have documented that the combination of acupuncture and MSC transplantation is superior to each procedure alone. The combination therapy can enhance the survival of MSCs, promote their differentiation into neurons, and facilitate their targeted migration to the spinal cord by stimulating the secretion of neurotrophic factors such as NT-3. Ultimately, these processes lead to the improvement of the microenvironment and generation of a functional neural network. Additionally, the differentiation of MSCs into neurons can overcome the shortage of endogenous NSCs in SCI patients. Therefore, acupuncture combined with MSC transplantation could become a novel and effective strategy for the treatment of SCI. This possibility needs to be verified by basic and clinical research.

## Figures and Tables

**Table 1:** Effect of acupuncture on the repair of spinal cord injury.

Study	Number of patients	Randomized, type of clinical trial	Acupuncture intervention	Control intervention	Effect indicators
[19]	100	Yes, RCT	EA (SI3 and B62; 75 Hz, 10 mV)	Usual SCI rehabilitation care	Neurologic and functional recovery↑, ASIA and FIM scores↑
[20]	48	Yes, RCT	Acupuncture (GV and the Back-shu acupoints according to the injury region)+electric pulsing stimulation (0.1~1 mA, 20 minutes)	Acupuncture (GB30, ST36, GB39, and SP6)+rehabilitation training	Modified Barthel index↑, function comprehensive assessment↑
[2]	1	No, a case report	Scalp acupuncture (DU24, DU19, DU18, DU21)	Not applicable	Motor function↑, neural plasticity↑
[21]	10	No, controlled trial	EA (LI4 and LI11; 5 Hz)	Not applicable	Activated C6 and C2 cervical spinal cord levels, functional MRI

Abbreviations: RCT: randomized controlled trial; SI3: *Houxi*; B62: *Shenmai*; ASIA: American Spinal Injury Association; GV: *Dumai*; GB30: *Huantiao*; ST36: *Zusanli*; GB39: *Xuanzhong*; SP6: *Sanyinjiao*; Ex-B2: *Jiaji*; LI4: *Hegu*; LI11: *Quchi*; DU24: *Shenting*; DU19: *Houding*; DU18: *Qiangjian*; DU21: *Qianding*.

**Table 2:** Mechanism of acupuncture on the repair of spinal cord injury.

Study	Acupuncture intervention	Control intervention	Effect indicators	Mechanism index
Neuroprotective effect of acupuncture				
[29]	EA (GV4; 2-100 Hz, 2.5 mA, 30 minutes)	EA (GV26)	Motor function↑, Basso-Beattie-Bresnahan (BBB) locomotor rating scale scores↑	Hydroxyl radical concentration↓, lipid peroxidation↓
[30]	EA (DU26, DU16; 2 Hz, 0.2 mA, 30 minutes) MA (DU26, DU16; 2 revolutions per second for 10 s, every 10 minutes, 30 minutes)	No treatment	Neuronal function recovery↑, antioxidation↑, anti-inflammation↑, antiapoptosis effects↑	IL-1*β*↓, IL-6↓, TNF-*α*↓
[31]	EA (GV6, GV9; 60 Hz for 1.05 s and 2 Hz for 2.85 s, ≤1 mA, 20 minutes, once every other day, 4 weeks)	No treatment	BBB functional↑	M1 (TNF-*α*↓, IL-1*β*↓, IL-6↓), M2 (IL-10↑, CD206↑), NT-3 expression↑, the polarization of M2 microglia/macrophages↑
[32]	Acupuncture (GV26, GB34; two spins/second, 30 s, 30 minutes, once a day, 2 weeks)	Not received any acupuncture treatment	Functional recovery↑	Caspase-3↓, p38 MAPK↓, resident microglia↓, TNF-*α*↓, IL-1*β*↓, IL-6↓, nitric oxide synthase↓, cycloxygenase-2↓, matrix metalloprotease-9↓
[33]	Elongated needle (BL54, ST28, CV6, CV3; 20-40 times/min, 1.5-3 V, 15 minutes)	No acupuncture stimulation	Decrease spinal injury↓, cell apoptosis↓	p-Akt and p-ERK1/2↑, Cyt c and caspase-3↓
[34]	EA (ST36, KI3; 60 Hz for 1.05 s and 2 Hz for 2.85 s, ≤2 mA, 20 minutes)	No electrical stimulation	Locomotor skills↑, ultrastructural features of the myelin sheath↑	Caspase-12↑, Cyt c↑, oligodendrocyte proliferation↑, oligodendrocyte death↓
[35]	EA (GV6, GV9; 60 Hz, 20 minutes)	No acupuncture stimulation	Functional recovery↑, tissue loss and neuronal apoptosis↓	Proapoptotic proteins (cleaved caspase-3/9↓ and cleaved PARP↓), antiapoptotic protein Bcl-2↑, miR-214↑
[36]	Elongated needle (BL54, ST28; 20–40beats/min, 1.5–3 V, 15 minutes)	Control group	Cell apoptosis↓	FAS → caspase-3 cascade↓
[37]	Elongated needle therapy (BL54, ST28; 2 Hz, 1-3 mA, 15 minutes)	Not received acupuncture treatment	BBB locomotor scale↑	PI3K/Akt and MAPK/ERK signaling pathways↑, Bax protein↓, Bcl-2↑, mitochondrial apoptosis pathway↓
[38]	EA (BL54, ST28, CV6, CV3; 20 Hz/40 Hz, 15 minutes)	Elongated needle EA	Promote repair↑	PI3K/Akt↑, ERK1/2↑, Cyt c↓, caspase-3↓
[29]	EA (GV4; 2.5 mA, 2-100 Hz, 30 minutes)	EA (GV26)	Motor function↑, BBB locomotor scale↑	Hydroxyl radical concentration↓, lipid peroxidation↓

Acupuncture modulates neural plasticity and promotes neural regeneration				
Wnt signaling pathway				
[39]	EA (GV14, GV4; 2 Hz, 1 mA, 20 minutes)	Not received EA treatment	Hindlimb motor functions↑, neuroprotective effects↑, proliferation and differentiation of neural stem cells↑	Wnt/*β*-catenin signaling pathway↑, proliferation and differentiation of neural stem cells↑
[40]	Fire needle acupuncture (T7, T8, T11, T12; 1/3 s, 3-5 mm, once a day)	Not treated by fire needle acupuncture	Lower limb locomotor function↑	Wnt/*β*-catenin↑, ERK↓, nestin↑, NSE↑, Gal-C↑, GFAP↓; Wnt-3a↑, GSK3*β*↑, *β*-catenin↑, ngn1↑, ERK1/2↓, cyclin D1 gene and protein↓
Notch signaling pathway				
[42]	EA (GV14, GV4; 2 Hz, 2 V, 30 minutes, once a day)	Without any treatment	Morphological recovery↑	Notch signaling pathway↓, promoting the proliferation of endogenous neural stem cells↑
[43]	EA (GB30, Ex-B05; 100 Hz/2 s and 2 Hz/2 s, 3 mA, 5 mm)	Not received EA treatment	Spontaneous regeneration↑, remyelination↑, recovery of function↑	BrdU(+)/NG2(+) cells↑, the proliferation of endogenous neural stem cells and oligodendrocytes↑
Rho/ROCK signaling				
[46]	EA (DU14, DU4, SP6, GB30, ST36, BL60; 4 Hz, 30 minutes, once a day, 7 days)	No acupuncture stimulation	Tissue repair and neurological functional recovery↑, BBB locomotor scale and inclined plane test scores↑	Neuronal apoptosis↓, decreases RhoA↓, Nogo-A mRNA↓
[47]	EA (GV3, GV14, ST36, BL32; 100 Hz for 1.5 ms and 2 Hz for 1.5 ms)	Blocking agent Y27632	Spinal cord tissue morphology↑, BBB score of lower limb movement function↑	Rho/ROCK signaling pathway↓, axonal growth and inflammatory reaction↓
[48]	EA (Ex-B2; 100 Hz, 30 minutes, once daily for 14 days and 28 days)	ROCK inhibitor groups	Hindlimb locomotor function↑	RhoA/ROCK signaling pathway↓ (RhoA↓, ROCK II↓, MLC proteins↓)
[49]	EA (GV3, GV14, ST36, BL32; 100 Hz for 1.5 ms and 2 Hz for 1.5 ms, 2 V, 20 minutes,14 days)	Blocking agent Y27632 EA+Y	Lower limb movement function↑	Nogo/NgR and Rho/ROCK signaling pathway↓ (mRNA and protein expression of Nogo-A↓, NgR↓, LINGO-1↓, RhoA and ROCK II↓)
[57]	EA (GV3, GV14, ST36, BL32; 2 Hz, 2 V, 20 minutes, 14 days)	Monosialoganglioside treatment	Hindlimb motor functions↑	Rho-A and Rho-associated kinase II (ROCK II)↓, Rho/ROCK signaling pathway↓
Neurotrophic factors				
[58]	EA (ST36, GB39, ST32, SP6; 2 Hz, 98 pulses per minute, 15 minutes, ST36 and GB39, first day, ST32 and SP6, second day, each pair of acupoints was stimulated on alternate days)	Not received EA treatment	Sensory functional↑	CNTF↓, p75-like apoptosis-(death domain protein↓, IGF-1↓, transforming growth factor-beta 2↓, FGF-4↓)
[53]	EA (ST36, GB39, ST32, SP6; alternating stimulus, 98 Hz, 30 minutes, the stimulating electrodes were changed and their polarity reversed after 15 minutes)	Not received EA treatment	Hindlimb locomotor and sensory functions↑	CNTF↑, FGF-2↑, TrkB mRNA↑; NGF, PDGF↓, TGF-*β*1↓, IGF-1↓, TrkA↓, TrkC mRNA↓
[50]	EA (GV1; 2 Hz, 2 mA, 20 minutes, once a day)	Not received EA treatment	BBB↑	NGF↑, BDNF↑
[59]	EA (ST36, GB39, ST32, SP6; 75 cycles/minute, 40-50 *μ*A, 30 minutes, once a day)	No treatment	BBB locomotor rating scale scores↑, motor neuron function↑	AChE activity↑, GDNF↑
[51]	EA (GV14, GV4; 2 Hz, 1 mA, 20 minutes)	No treatment	Motor function↑, neuronal function↑	NT-3↑
[52]	EA-2 group (GV20, GV16, GV14, GV4; GV14 and GV4, 2 Hz, 0.2 mA, 30 minutes, once every 2 days, 6 weeks)	EA-1 group	BBB locomotor rating scale scores↑, locomotor function↑	BDNF↑, NT-3↑
[54]	EA (GV14, GV4, GV7, GV5; alternating stimulus, 2 Hz, 10 minutes, 6 days EA-1 day interval-6 days EA)	No treatment	Movement function↑	BDNF↑, CREB↑
[38]	EA (BL54, ST28, CV6, CV3; 20 Hz/40 Hz)	Only performed a laminectomy	Promote repair↑	PI3K/Akt↑, ERK1/2↑, cytochrome c↓, caspase-3↓
[60]	EA (GV4, GV14; GV9, GV6; 2 Hz, 20 minutes)	Not received EA treatment	Hindlimb locomotor↑ and sensory functions↑	IGF-1↓, FGF-2↓, CNTF↓, PDGF↓, TGF-*β*1↓, TrkA↓, TrkB↓, TrkC↓, NTFs↑
[55]	EA (Ex-B2, 2 Hz; 3, 7, and 14 days)	Not received EA treatment	BBB locomotor scoring↑, hindlimb locomotor function↑	EGFR↓, GFAP↓, nerve axon regeneration↑
[56]	EA (Ex-B2, 2/100 Hz, 0.2 mA, 15 minutes)	Not received EA treatment	Locomotor function↑	L1↑, GFAP↑, (early phase)-(GFAP)↓, (later stages), nestin↑
[58]	EA (ST36, GB39, ST32, SP6; alternating stimulus, 2 Hz, 98 pulses/minute, 15 minutes, after the third day, stimulate every other day)	Not received EA treatment	Sensory functional↑	CNTF↓, p75-like apoptosis-(death domain protein↓, IGF-1↓, transforming growth factor-beta 2↓, FGF-4↓)

Abbreviation: GV4: *Mingmen*; GV26: *Shuigou*; BBB: Basso-Beattie-Bresnahan; DU16: *Fengfu*; IL-1*β*: interleukin-1*β*; IL-6: interleukin-6; TNF-*α*: tumor necrosis factor-*α*; GV6: *Jizhong*; GV9: *Zhiyang*; NT-3: neurotrophin-3; GB34: *Yanglingquan*; MAPK: mitogen-activated protein kinase; BL54: *Zhibian*; ST28: *Shuidao*; CV6: *Qihai*; CV3: *Zhongji*; Cyt c: cytochrome c; ST36: *Zusanli*; KI3: *Taixi*; GV4: *Mingmen*; GV14: *Dazhui*; SCI: spinal cord injury; ERK: extracellular signal-regulated kinase; GSK3*β*: glycogen synthase kinase 3*β*; GB30: *Huantiao*; Ex-B05: *Huatuojiaji*; BL32: *Ciliao*; ST32: *Futu*; GV1: *Changqiang*; NGF: nerve growth factor; BDNF: brain-derived neurotrophic factor; AChE: acetylcholinesterase; GDNF: glial cell line-derived neurotrophic factor; GV20: *Baihui*; GV7: *Zhongshu*; GV5: *Xuanshu*; Ex-B2: *Jiaji*; CREB: cAMP response element-binding; NTFs: neurotrophic factors; EGFR: epidermal growth factor receptor; GFAP: glial fibrillary acidic protein.

**Table 3:** Effects of the combination of acupuncture and MSC implantation on spinal cord injury and their mechanisms.

Study	Intervention & acupuncture parameters	Control intervention	Effect indicators	Comparison of effects between groups	Mechanism index
[78]	MSCs (1 × 10^6^ viable cells/mL), EA (Ex-B2; H1 = 2 Hz, H2 = 50 Hz, 20 minutes, 14 days), MSCs+EA	PBS group	Combined behavioral score↑	BMSC+acupuncture > acupuncture > BMSC > PBS	Differentiation of BMSC into neuronal cells↑, NSE↑, GFAP↑
[77]	MSCs (1 × 10^5^ viable cells/mL)+EA (GV1, GV2, GV6, GV9; 60 Hz for 1.05 s and 2 Hz for 2.85 s, ≤1 mA, 20 minutes), MSCs+EA	Sham-control Op-control	BBB locomotion test↑, differentiation of MSCs↑, regeneration of nerve fibers↑	MSCs+EA > EA > MSCs > control	GFAP↓, CSPGs↓
[81]	MSCs (5 × 10^5^ cells/mL)+EA (T9, T11; 39 A/h, 20 Hz, 15 minutes, twice a day), MSCs+EA	Normal group	Functional deficits↑, axonal regeneration↑	MSCs+EA > MSCs > EA > normal	GFAP↓, CSPGs↓, G-CSF↑, BDNF↑, VEGF↑, IL-6↑
[83]	MSCs (1 × 10^5^ cells/*μ*L, 5 *μ*L)+EA (GV1, GV2, GV6, GV9; 60 Hz for 1.05 s and 2 Hz for 2.85 s, once every other day, 7 weeks), MSCs+EA	Op-control	Axonal regeneration↑, partial locomotor functional↑	MSCs+EA > MSCs > EA > Op-control	NT-3↑, cAMP level↑, 5-HT↑, CGRP-positive nerve fibers↑
[85]	TrkC-MSCs (1 × 10^5^ cells/*μ*L, 5 *μ*L)+EA (GV6, GV9; 60 Hz for 1.05 s and 2 Hz for 2.85 s, ≤1 mA, 20 minutes), MSCs+EA	PBS group	Remyelination↑, functional↑	TrkC-MSCs+EA > MSCs+EA > TrkC-MSCs > MSCs	NT-3↑
[84]	MSCs (1 × 10^5^ cells/mL, 5 mL)+EA (GV9, GV6, GV2, GV1; 60 Hz for 1.05 s and 2 Hz for 2.85 s, 1 mA, 20 minutes); MSCs+EA	Normal group	BBB locomotion test↑	MSCs+EA > EA > MSCs > Op-control	Endogenous NT-3↑, 5-HT-positive nerve fibers↑

Abbreviations: BMSCs: bone marrow stromal cells; PBS group: PBS injection in the injured area; Ex-B2: *Jiaji*; PBS: phosphate-buffered saline; GV1: *Changqiang*; GV2: *Yaoshu*; GV6: *Jizhong*; GV9: *Zhiyang*; MSCs: mesenchymal stem cells; Sham-control: received a laminectomy without spinal cord transection; Op-control: operated control received a spinal cord transection only without any treatments; GFAP: glial fibrillary acidic protein; BBB: Basso-Beattie-Bresnahan; CSPGs: chondroitin sulfate proteoglycans; normal group: normal rats; G-CSF: granulocyte colony-stimulating factor; BDNF: brain-derived neurotrophic factor; VEGF: vascular endothelial growth factor; IL-6: interleukin-6; NT-3: neurotrophin-3.

## Data Availability

All data used during the study are available from the corresponding author by request “xuzhifangmsn@hotmail.com”.

## Abbreviations

ADL: Activity of daily living
ASIA: American Spinal Injury Association
BDNF: Brain-derived neurotrophic factor
CNSIs: Central nervous system injuries
CSPGs: Chondroitin sulfate proteoglycans
cAMP: Cyclic AMP
EA: Electroacupuncture
NTFs: Endogenous neurotrophic factors
ERK: Extracellular signal-regulated kinase
GFAP: Glial fibrillary acidic protein
GV: Governor Vessel
IL-1*β*: Interleukin-1*β*
IL-6: Interleukin-6
MSCs: Mesenchymal stem cells
MAPK: Mitogen-activated protein kinase
NGF: Nerve growth factor
NSCs: Neural stem cells
NG2: Neuron-glia antigen 2
NT-3: Neurotrophic factor-3
RNS: Reactive nitrogen species
ROS: Reactive oxygen species
ROCK: Rho/Rho kinase
SCI: Spinal cord injury
TNF-*α*: Tumor necrosis factor-*α*.
